# Study on the characteristics analysis and recognition method of vowels in patients with type Ⅱ diabetes

**DOI:** 10.3389/fdgth.2026.1739091

**Published:** 2026-05-18

**Authors:** Huawei Tao, Shuailong Zheng, Peng Li, Pengxu Jiang, Aiqin Li, Yunbing Meng

**Affiliations:** 1 Key Laboratory of Grain Information Processing and Control (Henan University of Technology), Ministry of Education, Zhengzhou, Henan, China; 2 Henan Key Laboratory of Grain Storage Information Intelligent Perception and Decision Making, Henan, China; 3 Institute for Complexity Science, Henan University of Technology, Zhengzhou, Henan, China; 4 Kaifeng Central Hospital, Kaifeng Municipal Health Commission, Kaifeng, Henan, China

**Keywords:** dynamic logistic regression, feature selection, intelligent recognition, type 2 diabetes, vowel features

## Abstract

Type 2 diabetes mellitus (T2DM) can induce impairments in vocal fold function and neural control, resulting in systematic changes in vowel articulation that may serve as objective biomarkers for speech-based disease detection. Traditional sentence-level approaches are susceptible to linguistic variability, limiting their ability to extract disease-specific acoustic features and reducing overall robustness. This study presents a vowel-based intelligent recognition framework for T2DM (VDT2) designed to capture stable and discriminative pathological speech features. The framework combines Lasso regression with recursive feature elimination to identify the most relevant acoustic indicators, and applies a dynamic logistic regression ensemble enhanced with an attention mechanism to strengthen feature representation. Experiments conducted on a self-constructed speech dataset demonstrate that VDT2 achieves a detection accuracy of 78%, outperforming conventional sentence-level methods. These findings highlight the potential of vowel-based analysis as a robust and non-invasive tool for T2DM detection.

## 1 Introduction

Diabetes has become one of the most widespread chronic diseases globally, with type 2 diabetes mellitus (T2DM) accounting for the majority of cases and significantly contributing to diabetes-related mortality. Due to its insidious onset, gradual progression, and complex complications, T2DM is frequently undiagnosed in its early stages; by the time of clinical diagnosis, the disease often has progressed to intermediate or advanced stages, thereby adversely affecting treatment efficacy (1, 2). Although current diagnostic methods—such as blood glucose monitoring (3, 4), urine analysis (5), and imaging examinations (6)—are well established, they have notable limitations, including invasiveness, high costs, and reliance on specialized equipment, which limit their accessibility in resource-constrained environments (7, 8).

In recent years, speech has gained prominence as a convenient, low-cost, and non-invasive signal carrier, with applications spanning multiple domains (9–15). Within the medical field, various physiological acoustic signals—such as respiratory, cardiac, and gastrointestinal sounds—encapsulate rich health-related information, serving as objective indicators of organ function and potential pathological changes. In individuals with diabetes, organ damage, metabolic disturbances, and related physiological dysfunctions may lead to alterations in speech characteristics, positioning speech as a promising external biomarker for disease manifestation. Furthermore, the widespread use of smart devices provides a technological foundation for contactless and affordable health monitoring, rendering speech-based diabetes detection an increasingly important area of research.

Recent medical studies have demonstrated that, in the early stages of diabetes, elevated blood glucose levels may reduce vocal fold elasticity and impair neural control, resulting in alterations in voice quality—such as decreased fundamental frequency (F0) and hoarseness. Importantly, these vocal changes often precede conventional clinical symptoms, offering significant potential for early diagnosis (16–20). At the intersection of linguistics and medicine, several investigations have explored the relationship between acoustic features in individuals with diabetes and their underlying pathological conditions. Chitkara et al. (21) analyzed the sustained vowel /a/ in 177 participants and reported that patients with T2DM exhibited significantly reduced jitter (frequency perturbation), amplitude perturbation quotient (APQ), and harmonic-to-noise ratio (HNR). Pinyopodjanard et al. (20) found that the F0 of sustained vowel phonation in female T2DM patients was significantly lower than in healthy controls. Gölaç et al. (22) further observed that patients with diabetic neuropathy showed pronounced abnormalities in maximum phonation time (MPT) and smoothed local amplitude perturbation (Slocal). Moreover, Suppakitjanusant et al. (23) reported that diabetic patients with fasting blood glucose levels above 200 mg/dL exhibited a significant reduction in the F0 of vowel /a/, and that HNR was strongly and negatively correlated with glycated hemoglobin (HbA1c) levels. Together, these findings underscore a potential association between acoustic biomarkers and diabetic status, providing a robust theoretical basis for the development of speech-based, non-invasive diagnostic methods.

With the rapid advancement of artificial intelligence and machine learning, several studies have explored the integration of individual speech features with machine learning models to enable intelligent recognition of diabetes. Spänig et al. (24) employed a neural network to non-invasively predict T2DM by combining voice interview data with demographic information, achieving an AUC of 0.703. Kaufman et al. (25) collected fixed-sentence speech via smartphones, extracted acoustic features, and developed gender-specific models that attained recognition accuracies exceeding 70%. Summoogum et al. (26) utilized a home voice assistant to analyze natural conversational speech for preliminary T2DM screening in noisy domestic environments. Elbéji et al. (27) applied standardized reading tasks combined with multi-acoustic feature fusion modeling to improve detection performance. However, these studies predominantly rely on sentence-level modeling, which presents two main limitations: first, the complexity of linguistic content and the variable use of vocal organs often confound semantic information with physiological characteristics, thereby reducing detection accuracy; second, related research (28) indicates that language differences restrict model generalizability, hindering the applicability across diverse populations and languages.

To address the aforementioned challenges, this study focuses on Mandarin lingual vowels. In collaboration with Kaifeng Central Hospital in Henan Province, we collected and constructed a speech database comprising six lingual vowels (/a/, /o/, /e/, /i/, /u/, /ü/) from patients with type 2 diabetes. Subsequently, we systematically analyzed the correlations between speech alterations and diabetic pathological states. An intelligent T2DM detection model based on vowel speech was developed, employing a combination of Lasso regression and recursive feature elimination (RFE) for effective feature selection. Finally, a dynamic logistic regression ensemble model enhanced with an attention mechanism was utilized to perform node-level weighting of input features for accurate prediction of diabetic status.

## 2 Materials and methods

### 2.1 Feature extraction

Table 1 summarizes the acoustic features currently employed internationally for speech analysis in diabetic patients (25). Additionally, the functional roles of each feature are presented alongside their variation patterns and potential influencing factors as reported in the literature. Research indicates that diabetes-induced dysfunctions across multiple organs-including laryngeal muscles, vocal cord mucosa, the respiratory system, and neural control pathways-are significantly associated with alterations in speech acoustic parameters. This association offers a valuable diagnostic reference for diabetes detection based on vowel speech analysis.

**Table 1:** Feature analysis.

Feature	Feature explanation	Summary of relevant references
MeanF0 StdevF0	The fundamental frequency of vocal cord vibration, which determines the pitch of speech.	The significant decrease in MeanF0 in /a/ usually reflects the weakening of laryngeal muscle strength (22) The significant decrease in /a/ reflects the weakening of laryngeal muscle strength or vocal cord edema (25); the significant decrease in StdevF0 in /a/ is mainly caused by increased blood sugar levels (23) The significant decrease in /a/ is caused by the increased periodic stability of vocal cord vibration (25);
Localjitter Localabsjitter Rapjitter Ppq5jitter	Small temporal fluctuations in the fundamental frequency cycle, associated with vocal cord control disorders or pathology.	There is no significant difference in the values of /a/ in Localjitter, which may indicate problems such as abnormal vocal cord mucosa (21) The values of /a/ are significantly reduced (female), mainly due to reduced vocal cord tension (22) The values of /a/ are significantly increased, which may be caused by diabetic neuropathy (23); the values of /a/ in Localabsjitter are significantly reduced (female), mainly due to reduced vocal cord tension (21) The values of /a/ are significantly increased, mainly due to slight changes in the vocal cord cycle (22) The values of /a/ are significantly increased, indicating the presence of vocal cord mucosal damage or abnormal nerve control (25); the values of /a/ in Rapjitter are significantly reduced, mainly due to decreased laryngeal muscle coordination (23) The values of /a/ are significantly reduced, which may be due to vocal cord edema or improved muscle coordination (25); the values of /a/ in Ppq5jitter are significantly increased, mainly related to laryngeal muscle fatigue related to diabetes (25);
Meanlnten Stdevlnten	The intensity or loudness of a speech signal is directly related to the degree of vocal cord closure and airflow pressure.	A significant decrease/increase in Meanlnten/Stdevlnten in /a/ is due to diabetes-related decreased laryngeal muscle strength, abnormal respiratory regulation, laryngeal muscle control disorder, or mucosal lesions (25);
HNR	The energy ratio of periodic harmonic components to non-periodic noise components in a speech signal is often seen in breathy voices, hoarseness, or vocal cord injury.	The HNR value in /a/ is significantly reduced, which is due to the reduction of noise caused by the incomplete closure of the vocal cords (21). The HNR value in /a/ is significantly reduced, indicating that the stability of the vocal cord vibration period is affected by blood sugar control (23).
Localshimmer Localdbshimmer Apq3shimmer Apq5shimmer Apq11shimmer	Small fluctuations in the amplitude of the vocal cord vibration cycle, that is, differences in peak amplitude between adjacent cycles, can indicate vocal cord injury or abnormal neuromuscular control.	The value of Localshimmer in /a/ is significantly increased, indicating that the amplitude stability of the vocal cords is impaired (20) The value of /a/ is significantly increased, indicating that there is an abnormality in laryngeal muscle control (22) The significant increase in /a/ may be due to a decrease in laryngeal muscle strength or changes in mucosal elasticity (25); the value of Localdbshimmer in /a/ is significantly increased, indicating incomplete vocal cord closure or mucosal lesions (22) The significant increase in /a/ indicates the presence of incomplete vocal cord closure or mucosal lesions (25); the value of Apq3shimmer/Apq5shimmer/Apq11shimmer in /a/ is significantly decreased, resulting from abnormal respiratory airflow control ability (21) The value of /a/ is decreased in diabetic patients, mainly due to decreased respiratory control ability (20) The mechanism of significant decrease/increase/increase in/a/ involves vocal cord edema, decreased respiratory control ability or diabetic laryngeal muscle control disorder (25).

### 2.2 Design of an intelligent recognition method for type 2 diabetes based on vowel speech

**Figure 1:**
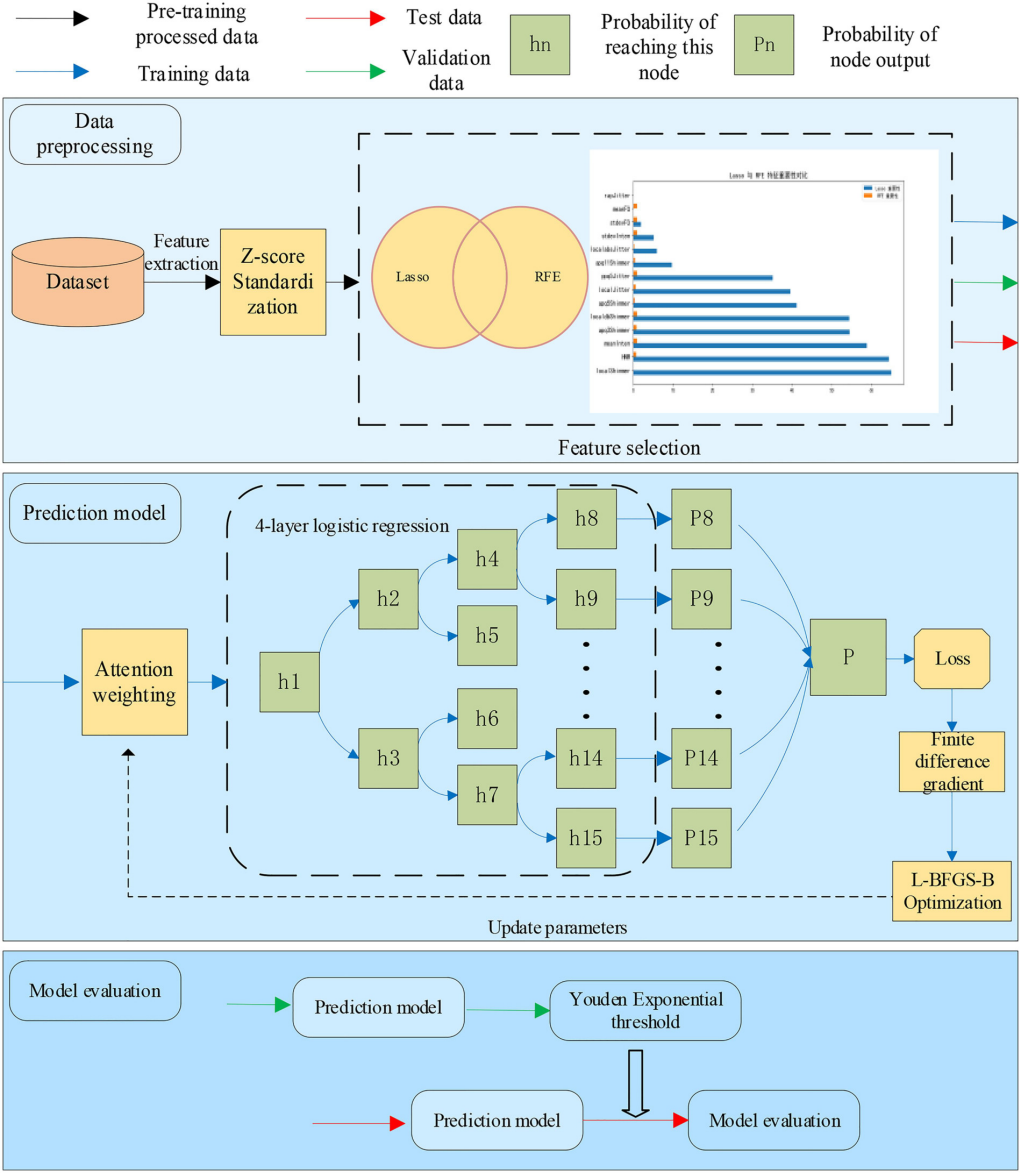
Model system block diagram.

This study proposes an intelligent method based on vowel-based speech for identifying type 2 diabetes. The model employs a fusion of Lasso regression and recursive feature elimination (RFE) for effective feature selection. Subsequently, a dynamic logistic regression ensemble model combined with an attention mechanism performs node-level weighting of input features, adaptively adjusting the contribution of each acoustic feature in different decision paths. This hierarchical decision-making process ultimately enables accurate identification of diabetic patients. The model framework is shown in Figure 1, and the model comprises the following specific modules:

#### 1. Feature preprocessing

Extract acoustic features such as MeanF0, StdevF0, MeanInt, StdevInt, LocalJitter, LocalAbsJitter, and RapJitter. Then, normalize the feature matrix to eliminate differences in feature dimensions. The normalization formula is shown in (1).

$$X_{\mathrm{norm}}=\frac{X-\mu }{\sigma }$$ (1)

where *X* is the original feature matrix, *μ* and *σ* are the mean and standard deviation of the training set, respectively.

#### 2. Feature selection

**Figure 2:**
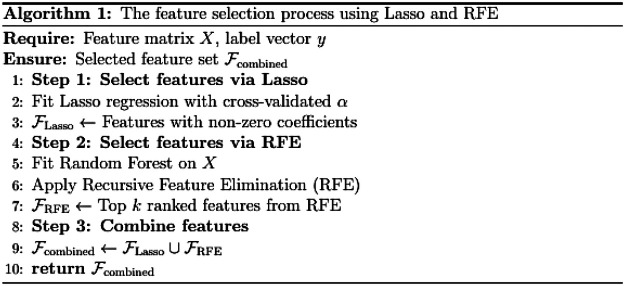
Feature selection algorithm process.

We developed a feature selection method that integrates Lasso regression with recursive feature elimination (RFE). This approach effectively removes redundant information while preserving core features. Lasso performs sparse selection, enhancing sensitivity to features with weak individual effects that interact synergistically with other variables. RFE recursively trains the model and progressively eliminates the least influential features. The combination of these methods enables a more comprehensive screening of key features. The detailed algorithmic workflow is illustrated in Figure 2.

#### 3. Dynamic logistic regression ensemble model with attention mechanism

**Figure 3:**
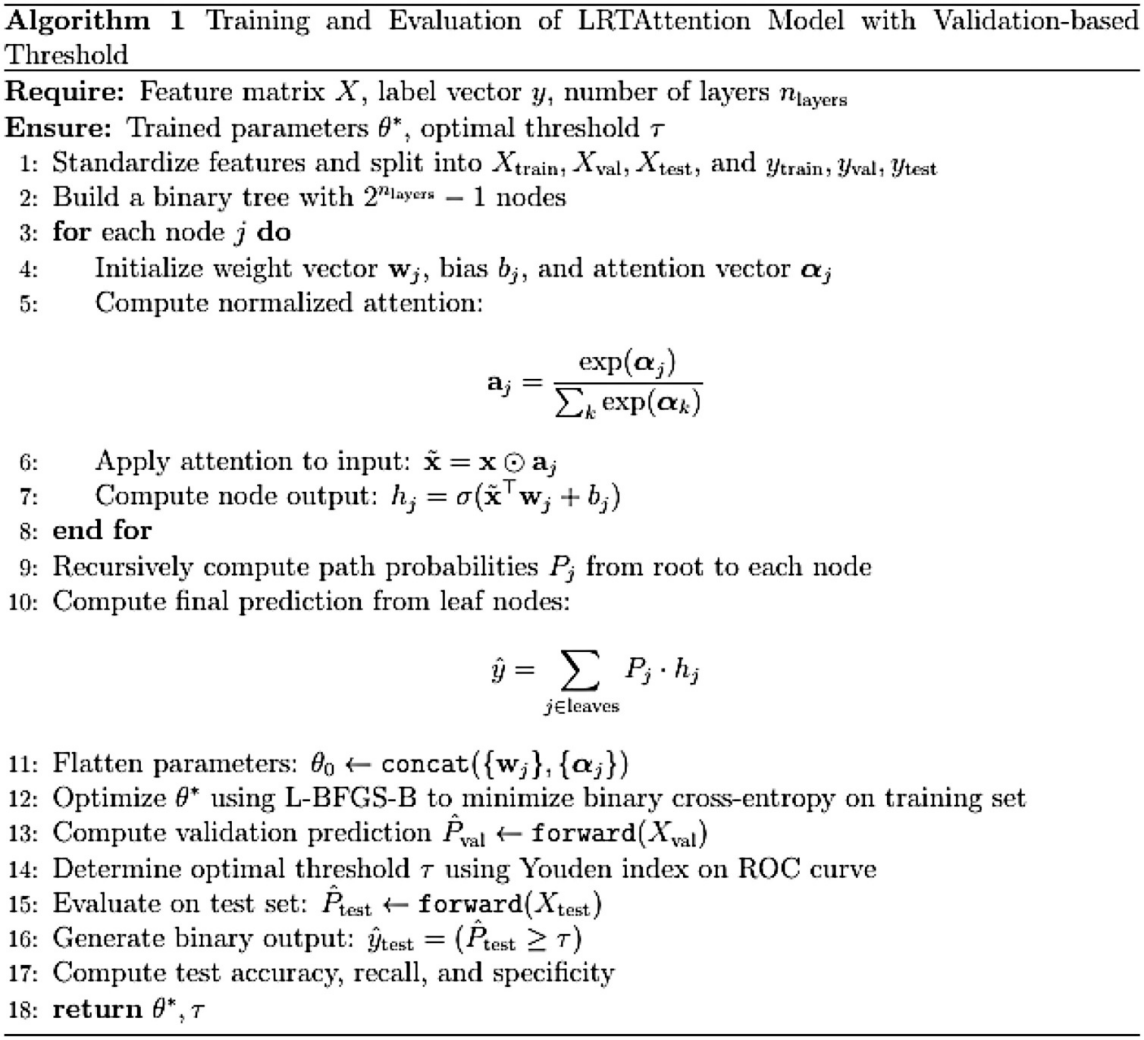
Model-specific process.

This model employs logistic regression units arranged in a complete binary tree structure, with each internal node functioning as a binary classifier. Learnable attention parameters are incorporated to perform node-level weighting of input features, adaptively modulating the contribution of each acoustic feature along different decision paths and enabling a hierarchical decision-making process. The detailed procedure is illustrated in Figure 3.

## 3 Results

### 3.1 Experimental setup and evaluation criteria

Database partitioning: The dataset was divided into training, validation, and test sets in a ratio of 6:2:2. To avoid potential data leakage, the partitioning was performed at the subject level rather than the sample level. Specifically, all speech recordings from the same participant were assigned exclusively to one subset, ensuring that no subject appeared in more than one of the training, validation, or test sets.

Parameter settings: The learning rate was set to 1 × 10^−5^, the maximum number of iterations of the optimization algorithm was 200, the number of tree-structured logistic regression layers was 4, and the random seed was set to 42.

Optimization method: The L-BFGS-B optimizer was adopted, and both the weight parameter w and attention parameter a were initialized using a Gaussian distribution.

Evaluation criteria: The model performance was evaluated using Accuracy, Recall, Specificity, and AUC (29).

### 3.2 Chinese tongue-front vowel phonetic database

**Figure 4:**
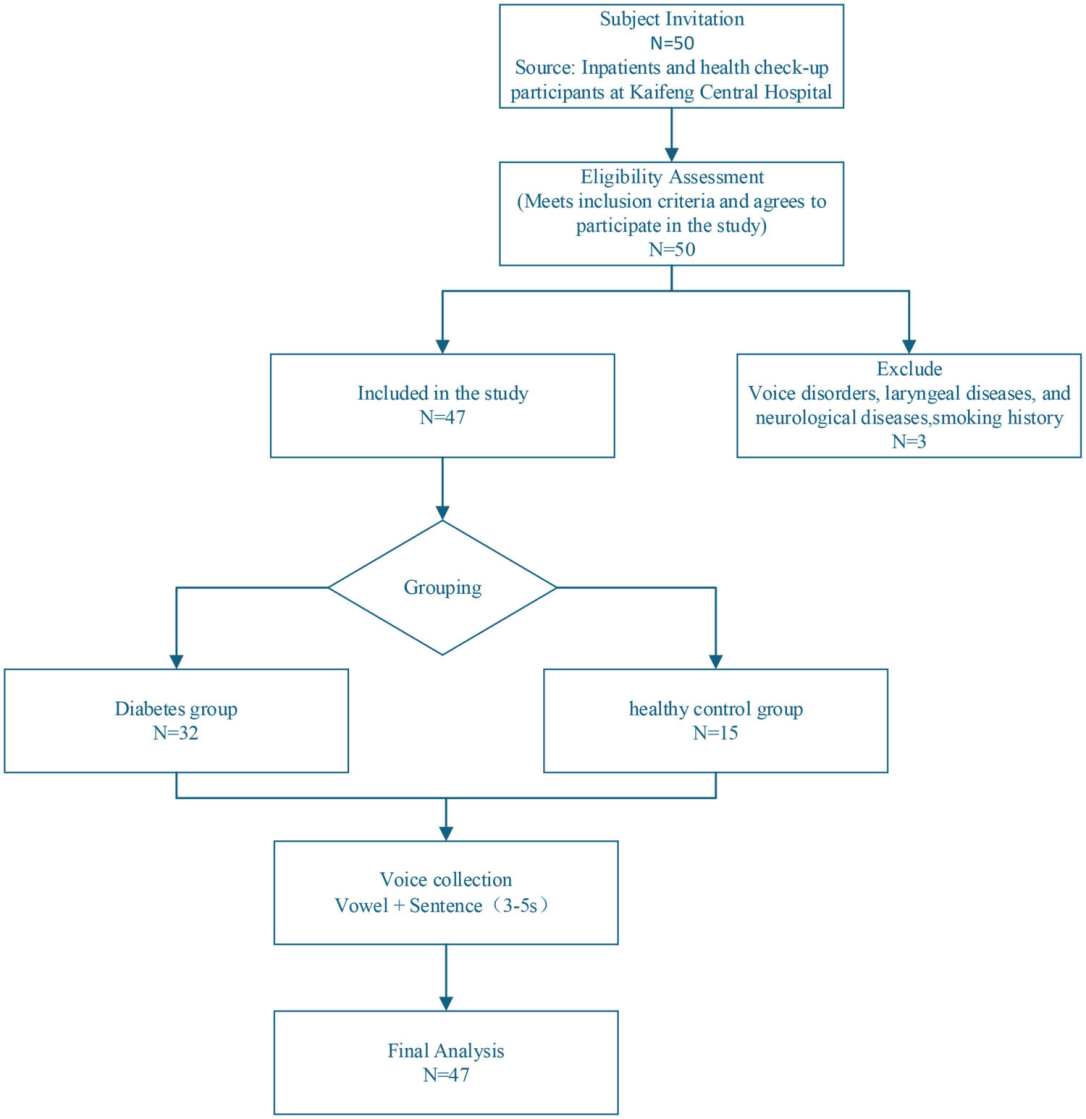
Subject screening process.

China bears one of the highest diabetes burdens worldwide, with over 118 million individuals diagnosed with type 2 diabetes mellitus (T2DM), accounting for 22% of the global total. To investigate intelligent methods for T2DM detection, this study, conducted in Mandarin, collaborated with Kaifeng Central Hospital in Henan Province to collect speech data from 32 T2DM patients and 15 healthy controls. The speech dataset focused on five basic lingual vowels (/a/, /o/, /e/, /i/, /u/) commonly found across various United Nations languages to ensure cross-linguistic applicability. Additionally, the unique Chinese vowel /ü/ was included to enhance linguistic diversity and representativeness. Ultimately, a T2DM speech database comprising six vowel categories was established. Figure 4 illustrates the recruitment, screening, and grouping process of the participants in this study. All participants were inpatients and healthy individuals undergoing physical examinations at Kaifeng Central Hospital. After eligibility assessment and exclusion criteria, a total of 47 participants were included, with 32 in the diabetes group and 15 in the healthy control group. All participants completed a standardized voice data collection process, and the final data were used for subsequent feature analysis and model construction.

Basic demographic characteristics of the participants, including age and sex distribution, were summarized and are presented in Table 2. The two groups showed no significant differences in these baseline characteristics, indicating comparability between the T2DM and control groups. Although additional clinical indicators, such as body mass index (BMI) and glycated hemoglobin (HbA1c), were available for a subset of participants, these variables were not consistently recorded across the entire cohort. Therefore, they were not included in the statistical analysis to ensure data consistency and avoid potential bias.

**Table 2:** Basic demographic characteristics.

Variable	Diabetes group	Control group
Age	40–70	40–70
Sex (Male/Female)	M/F	M/F
BMI	—	—
duration of diabetes (years)	4–10	0
HbA1c	—	—
related complications	None	None
smoking history	None	None
language/accent	Mandarin	Mandarin

**Table 3:** Acoustic characteristics of type 2 diabetes patients.

feature	T2DM					
	/a/	/o/	/e/	/i/	/u/	/ü/
meanF0	155.30 ± 33.32	160.09 ± 32.92	166.67 ± 33.55	174.34 ± 37.99	177.14 ± 37.97	176.97 ± 36.19
stdevF0	19.65 ± 19.47	18.08 ± 17.37	16.56 ± 19.63	21.05 ± 20.45	21.40 ± 22.67	16.30 ± 15.49
meanInten	64.41 ± 12.65	67.27 ± 10.53	68.42 ± 9.84	65.78 ± 8.53	68.01 ± 9.73	66.82 ± 8.91
stdevInten	16.23 ± 5.29	16.34 ± 5.35	16.36 ± 5.73	14.75 ± 4.56	15.195 ± 6.348	14.32 ± 5.73
HNR	14.469 ± 4.03	18.52 ± 4.59	19.29 ± 4.34	17.70 ± 4.46	21.797 ± 5.075	19.70 ± 4.706
localShimmer	0.062 ± 0.033	0.05 ± 0.04	0.036 ± 0.03	0.052 ± 0.036	0.043 ± 0.032	0.053 ± 0.037
localdbShimmer	0.686 ± 0.370	0.53 ± 0.36	0.438 ± 0.325	0.563 ± 0.346	0.455 ± 0.309	0.540 ± 0.336
apq3Shimmer	0.029 ± 0.015	0.02 ± 0.02	0.018 ± 0.016	0.025 ± 0.017	0.022 ± 0.017	0.026 ± 0.018
apq5Shimmer	0.038 ± 0.022	0.03 ± 0.02	0.022 ± 0.024	0.032 ± 0.023	0.027 ± 0.022	0.033 ± 0.024
apq11Shimmer	0.057 ± 0.042	0.04 ± 0.06	0.030 ± 0.032	0.047 ± 0.053	0.035 ± 0.028	0.043 ± 0.034
localJitter	0.011 ± 0.008	0.009 ± 0.007	0.009 ± 0.007	0.009 ± 0.007	0.008 ± 0.006	0.009 ± 0.007
localabsJitter	0.000 ± 0.000	0.000 ± 0.000	0.000 ± 0.000	0.000 ± 0.000	0.000 ± 0.000	0.000 ± 0.000
rapJitter	0.005 ± 0.004	0.004 ± 0.004	0.004 ± 0.004	0.004 ± 0.004	0.004 ± 0.003	0.004 ± 0.004
ppq5Jitter	0.006 ± 0.004	0.005 ± 0.004	0.005 ± 0.004	0.004 ± 0.004	0.004 ± 0.003	0.004 ± 0.004

**Table 4:** Normal human acoustic characteristics data.

feature	ND					
	/a/	/o/	/e/	/i/	/u/	/ü/
meanF0	180.13 ± 44.486	188.92 ± 41.44	191.54 ± 43.19	174.34 ± 37.99	199.13 ± 45.58	194.41 ± 44.05
stdevF0	14.979 ± 15.883	16.926 ± 17.746	19.190 ± 18.172	21.05 ± 20.45	21.359 ± 19.429	23.583 ± 19.634
meanInten	68.749 ± 12.290	71.217 ± 9.130	69.494 ± 9.392	65.78 ± 8.53	69.126 ± 8.754	68.144 ± 9.622
stdevInten	14.709 ± 6.247	16.349 ± 6.862	16.959 ± 5.867	14.75 ± 4.56	16.068 ± 7.203	14.684 ± 6.257
HNR	17.180 ± 3.248	20.507 ± 3.340	20.509 ± 2.936	17.70 ± 4.46	23.511 ± 5.042	19.889 ± 6.099
localShimmer	0.051 ± 0.032	0.036 ± 0.020	0.036 ± 0.020	0.052 ± 0.036	0.039 ± 0.032	0.057 ± 0.047
localdbShimmer	0.526 ± 0.321	0.425 ± 0.254	0.417 ± 0.238	0.563 ± 0.346	0.427 ± 0.305	0.576 ± 0.415
apq3Shimmer	0.025 ± 0.016	0.017 ± 0.009	0.017 ± 0.010	0.025 ± 0.017	0.019 ± 0.017	0.029 ± 0.025
apq5Shimmer	0.031 ± 0.020	0.021 ± 0.011	0.021 ± 0.012	0.032 ± 0.023	0.024 ± 0.021	0.037 ± 0.033
apq11Shimmer	0.044 ± 0.029	0.031 ± 0.019	0.031 ± 0.021	0.047 ± 0.053	0.031 ± 0.027	0.046 ± 0.044
localJitter	0.007 ± 0.006	0.007 ± 0.005	0.006 ± 0.004	0.009 ± 0.007	0.006 ± 0.003	0.009 ± 0.007
localabsJitter	0.000 ± 0.000	0.000 ± 0.000	0.000 ± 0.000	0.000 ± 0.000	0.000 ± 0.000	0.000 ± 0.000
rapJitter	0.004 ± 0.003	0.004 ± 0.003	0.003 ± 0.002	0.004 ± 0.004	0.003 ± 0.002	0.005 ± 0.004
ppq5Jitter	0.004 ± 0.003	0.004 ± 0.003	0.003 ± 0.002	0.004 ± 0.004	0.003 ± 0.002	0.005 ± 0.004

**Figure 5:**
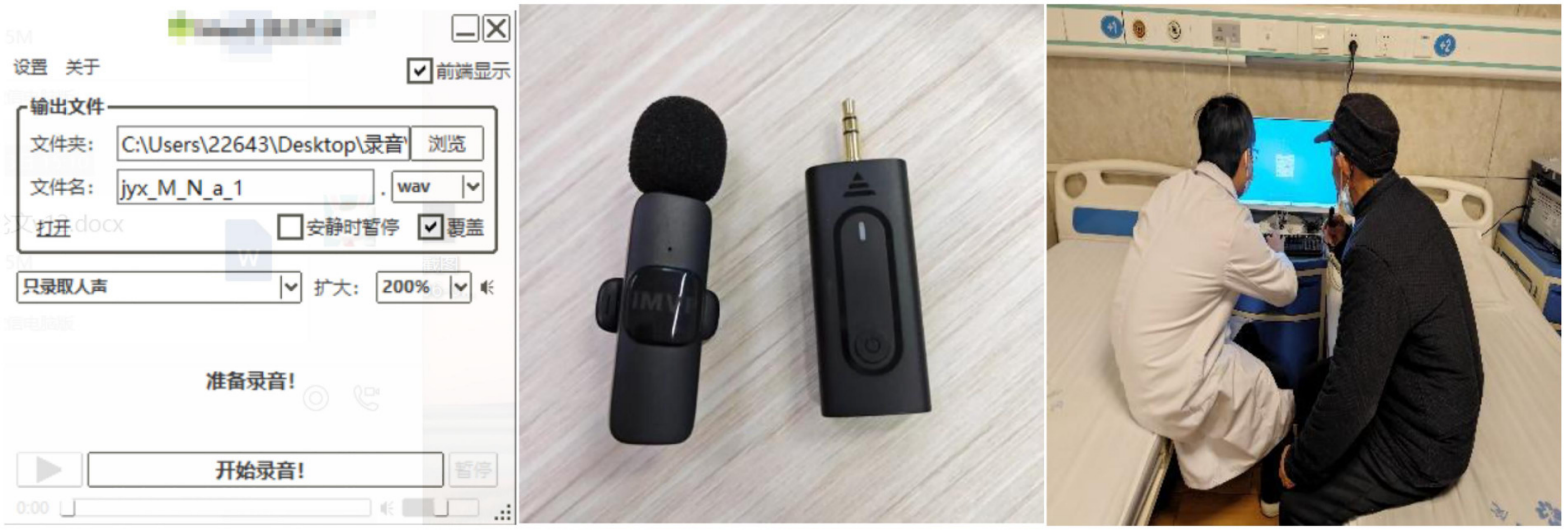
Recording software, equipment, and scenarios.

After completing the initial recordings, data cleaning was performed to remove incomplete or heavily noisy audio samples, ultimately retaining 542 high-quality vow-el speech data. Furthermore, to expand the analysis dimension, 86 additional speech samples containing complete sentences were collected, resulting in a total of 628 speech data. The recording equipment and environment are shown in Figure 5, and the relevant recording requirements are listed below:

1. Data recording requirements: vowels (/a/, /o/, /e/, /i/, /u/, /ü/), sentence: Mom has a green jade pot, each pronunciation is recorded twice, the duration is 4 s;
2. Personnel ratio: 32 diabetic patients, 15 normal people, the male-female ratio is as close to 1:1 as possible;
3. Recording location: Kaifeng Central Hospital;
4. Recording equipment and parameters:
  1. Noise-reduction lavalier microphone (Shenzhen Zhuochuang Network Tech-nology Co., Ltd.);
  2. Sampling frequency 44.1 KHZ, voice data is dual-channel;
  3. Indoor quiet scene;
5. Naming format: name_M_T/N/sen_a_1.wav;
6. Exclusion criteria: neurological/language disorders, respiratory diseases, acute infections (colds);

### 3.3 Feature validity analysis experiment

To further verify the feasibility of using speech features for diabetes detection, this study used independent samples *t*-tests to assess the statistical significance of various features between diabetic patients and healthy controls, as shown in Tables 3 and 4. Bonferroni correction was applied to the p-values ​​in the speech parameter statistical analysis to control for multiple comparisons. According to Table 5, four features showed significant differences (*p* < 0.05): mean fundamental frequency (meanF0), harmonic noise ratio (HNR), local jitter, and local absolute jitter. Subsequently, the discriminative power of each feature and its combinations was analyzed from the perspectives of information content and feature clustering to verify their potential application value in diabetes detection.

**Table 5:** Acoustic analysis.

Feature	*P*	Bonferroni correction p	Cohen's_*d*
meanF0	0.000000004	0.00000006	0.59449
stdevF0	0.7601	1	0.02811
meanInten	0.0182	0.254326	0.21799
stdevInten	0.8233	1	0.02151
HNR	0.00006	0.000887	0.36093
localShimmer	0.1351	1	−0.1377
localdbShimmer	0.05755	0.8057421	−0.17509
apq3Shimmer	0.14350	1	−0.13478
apq5Shimmer	0.15379	1	−0.13141
apq11Shimmer	0.11038	1	−0.14712
localJitter	0.00252	0.035362	−0.25461
localabsJitter	0.00003	0.000421	−0.34877
rapJitter	0.05189	0.7265139	−0.17925
ppq5Jitter	0.03997	0.5596049	−0.18944

#### 3.3.1 Feature differentiation analysis experiment based on information theory

**Figure 6:**
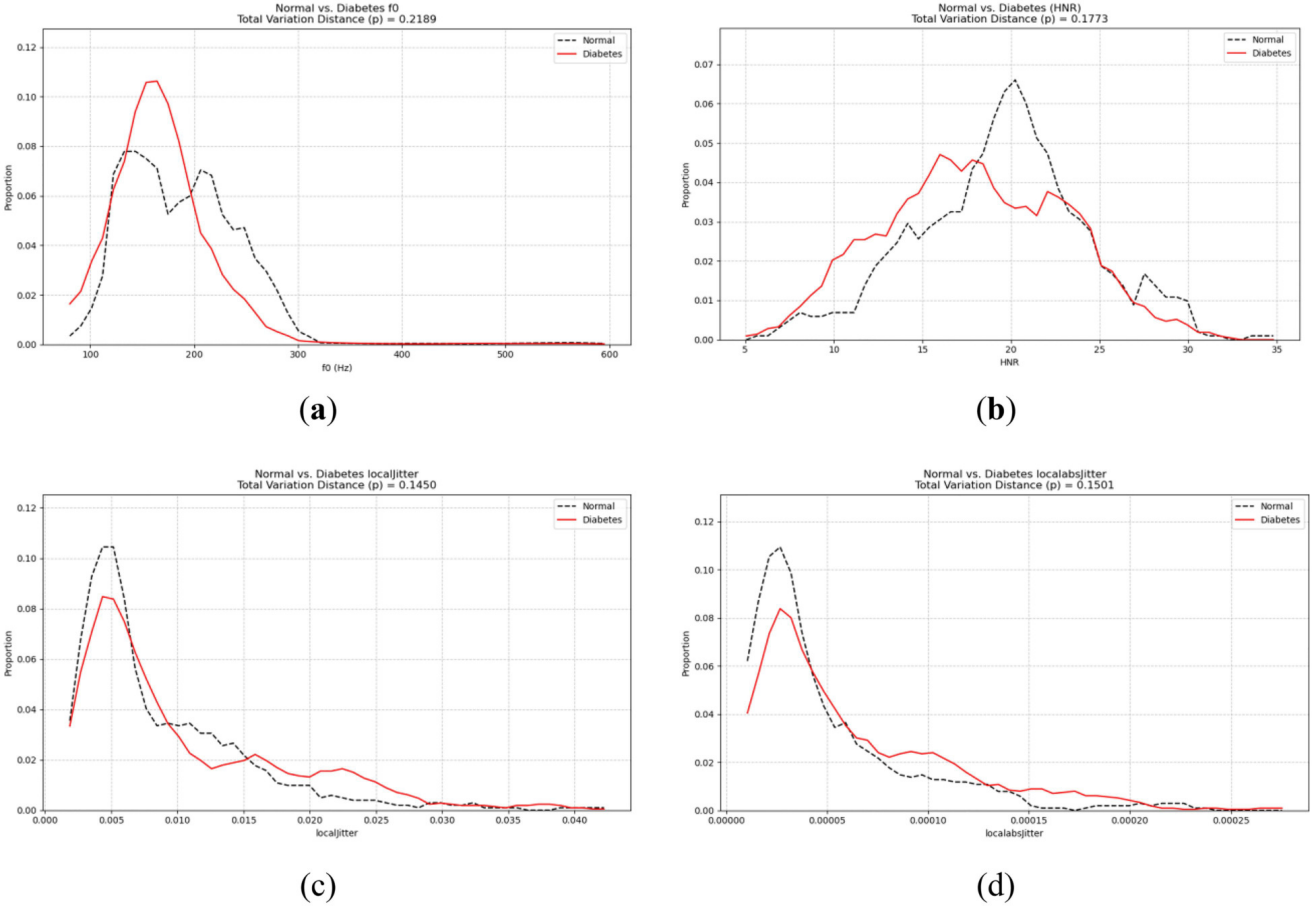
Distribution of **(a)** mean standard deviation (meanF0), **(b)** harmonic noise ratio (HNR), **(c)** local jitter, **(d)** local absolute jitter (localdbjitter).

The amount of information—such as information gain or entropy reduction—quantifies a feature's discriminative power in a classification task, specifically its ability to reduce uncertainty regarding the target variable (i.e., the presence or absence of diabetes). The greater the information content, the more effective the feature is at distinguishing between categories and the more discriminative information it carries. To assess the role of speech features in diabetes recognition, this study performed a systematic statistical analysis of features extracted from both healthy individuals and diabetic patients. Specifically, feature values were partitioned into equal intervals based on their minimum and maximum ranges, with the number and proportion of samples within each interval calculated to generate feature distribution curves, as illustrated in Figure 6. The normal speech feature distribution is (black curve), and the diabetic speech feature distribution is (red curve), so the difference between the two is $p=\frac{\int_{a}^{b}|\;f_{N}(x)-f_{T}(x)|dx}{2}$.

The feature distribution plots demonstrate that diabetic participants exhibit noticeable alterations in several acoustic parameters compared to healthy individuals. Specifically, the fundamental frequency (f0) distributions tend to shift toward higher values and display broader variability, suggesting increased irregularity in vocal fold vibration patterns. The jitter-related measures (e.g., localJitter and localAbsJitter) are elevated in diabetic speech, indicating a decline in frequency stability and periodic consistency of phonation. Moreover, amplitude perturbation parameters, such as localdbShimmer, show higher fluctuation amplitudes, reflecting weakened control over glottal closure and subglottal pressure regulation. Collectively, these deviations imply that diabetes impairs the fine motor control required for stable vocal fold oscillation and precise respiratory–laryngeal coordination. The underlying physiological mechanisms likely involve diabetes-induced neuropathy, reduced elasticity of the vocal folds, and compromised respiratory function, which jointly contribute to observable acoustic degradation in vowel production.

The above acoustic parameters form observable differences between groups, and this difference can be quantified by the amount of information. Diabetes detection is usually divided into two states: normal and diseased. Therefore, this paper refers to the existing voice detection information model (30) to construct a diabetes voice information model (29, 31). The specific formulas (2), (3), and (4) are shown below.

$$I_{\mathrm{same}}=\mathrm{lo}g_{2}(2)=1\;\mathrm{bit}$$ (2)

$$I_{\mathrm{diabetes}}=\mathrm{lo}g_{2}(14\times 2\times N)=\mathrm{lo}g_{2}\left(N\right)+4.81\;\mathrm{bit}$$ (3)

$$\eta =\frac{I_{\mathrm{same}}}{I_{\mathrm{diabetes}}}=\frac{1}{\log(N)+4.81}\;\mathrm{bit}$$ (4)

where $I_{\mathrm{same}}$ is the self-information about whether someone has diabetes, and $I_{\mathrm{diabetes}}$ is the self-information provided by each feature for each person.

**Figure 7:**
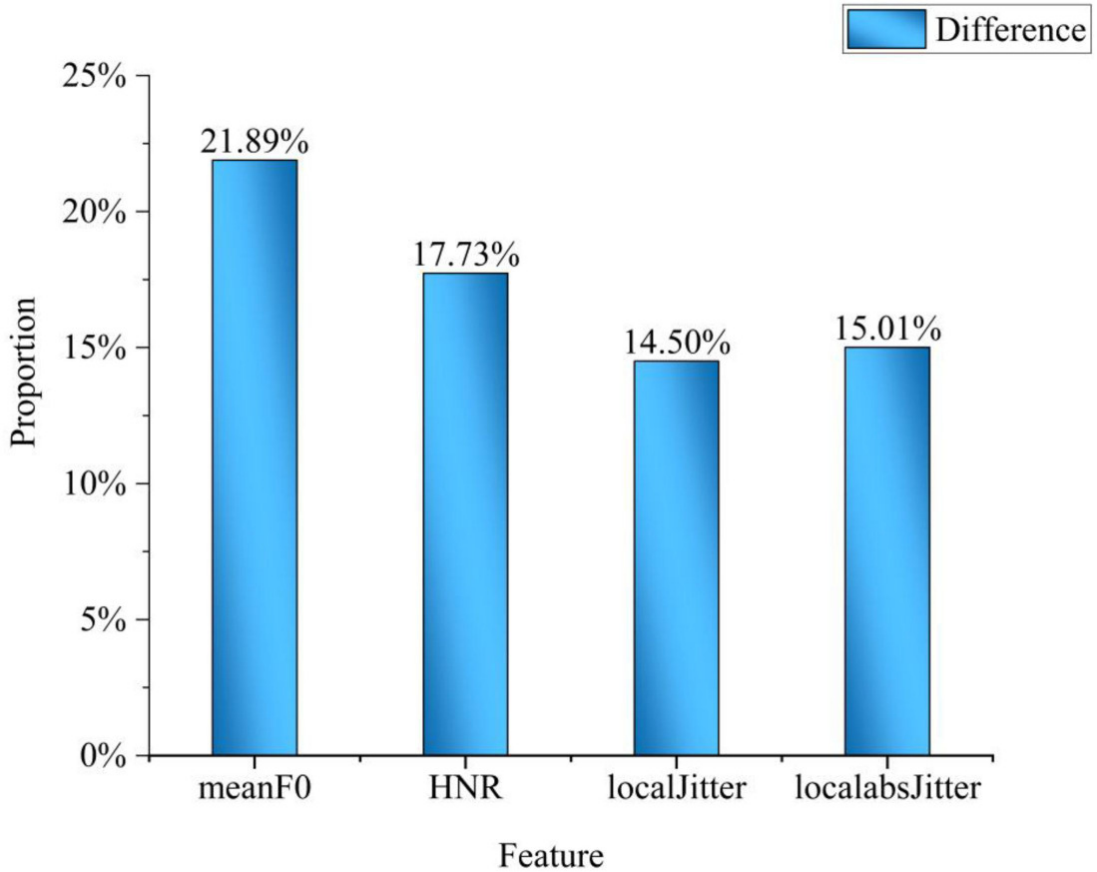
Difference statistics.

Furthermore, this paper statistically analyzed the differences in the feature distribution curves, as shown in Figure 7, and calculated the difference threshold based on the information content model. For individual analysis, when *N* = 1, $\eta =\frac{1}{\mathrm{lo}g_{2}(1)+4.81}=20.79\text{\%}$; for group analysis, when *N* = 30, η=10.28%, and when *N* = 50, η=9.57%. Figure 6 shows that most feature parameter distributions meet the basic difference threshold, indicating that the speech features do carry physiological information related to diabetes.

#### 3.3.2 Validity analysis experiment based on feature clustering

**Figure 8:**
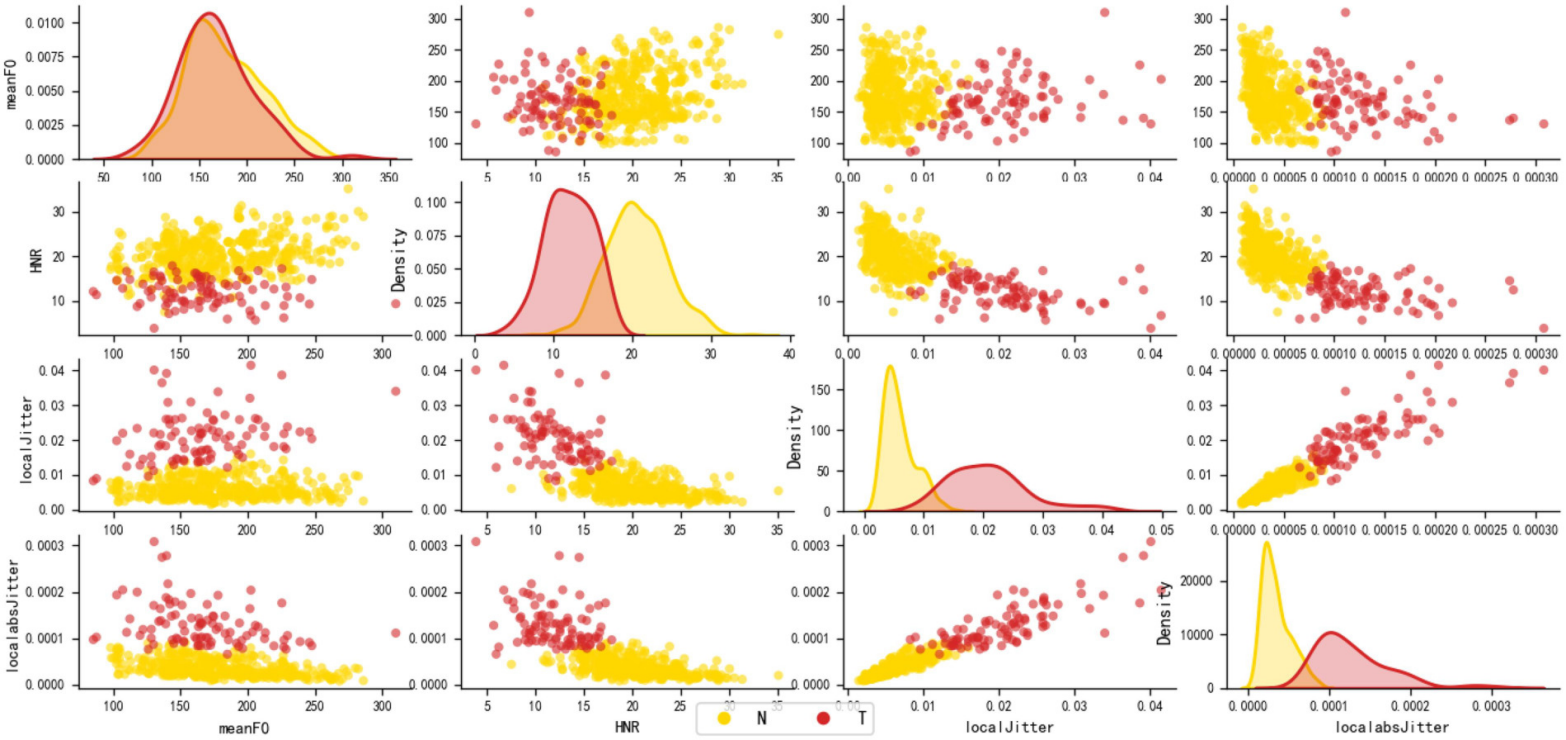
Cluster analysis.

Building on the analysis of feature distribution differences, Figure 8 further visualizes the recognition performance of the selected acoustic features through kernel density estimation (KDE) and scatter plots of pairwise feature combinations. The KDE results reveal significant distinctions in the probability density functions of diabetic and healthy groups across individual feature dimensions in terms of peak location, shape, and distribution width, with minimal overlap. This indicates that these features possess strong discriminative power even within a single-dimensional space. Moreover, scatter plots depicting pairwise feature relationships demonstrate a co-discriminative effect among acoustic parameters. Samples from different categories exhibit clear clustering patterns in the feature space, reflecting robust inter-class separability. These findings not only visually confirm the efficacy of the feature selection process but also provide theoretical support and a data-driven foundation for the subsequent development of a diabetic speech recognition model based on multidimensional joint features.

### 3.4 Feature selection experiments

**Figure 9:**
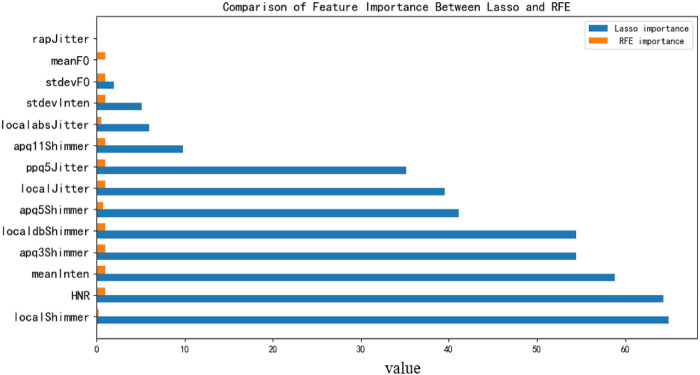
Feature importance comparison**.**

Based on information-theoretic analysis and feature clustering visualization results, this study constructs a machine learning-based classification framework to further explore the discriminative ability of speech features in diabetes detection. To enhance feature representation and robustness, two complementary feature selection methods—Lasso regression and Recursive Feature Elimination (RFE)—were integrated to construct a joint feature subspace for subsequent model training and evaluation. Figure 9 presents the quantitative ranking of feature importance obtained from both methods.

**Figure 10:**
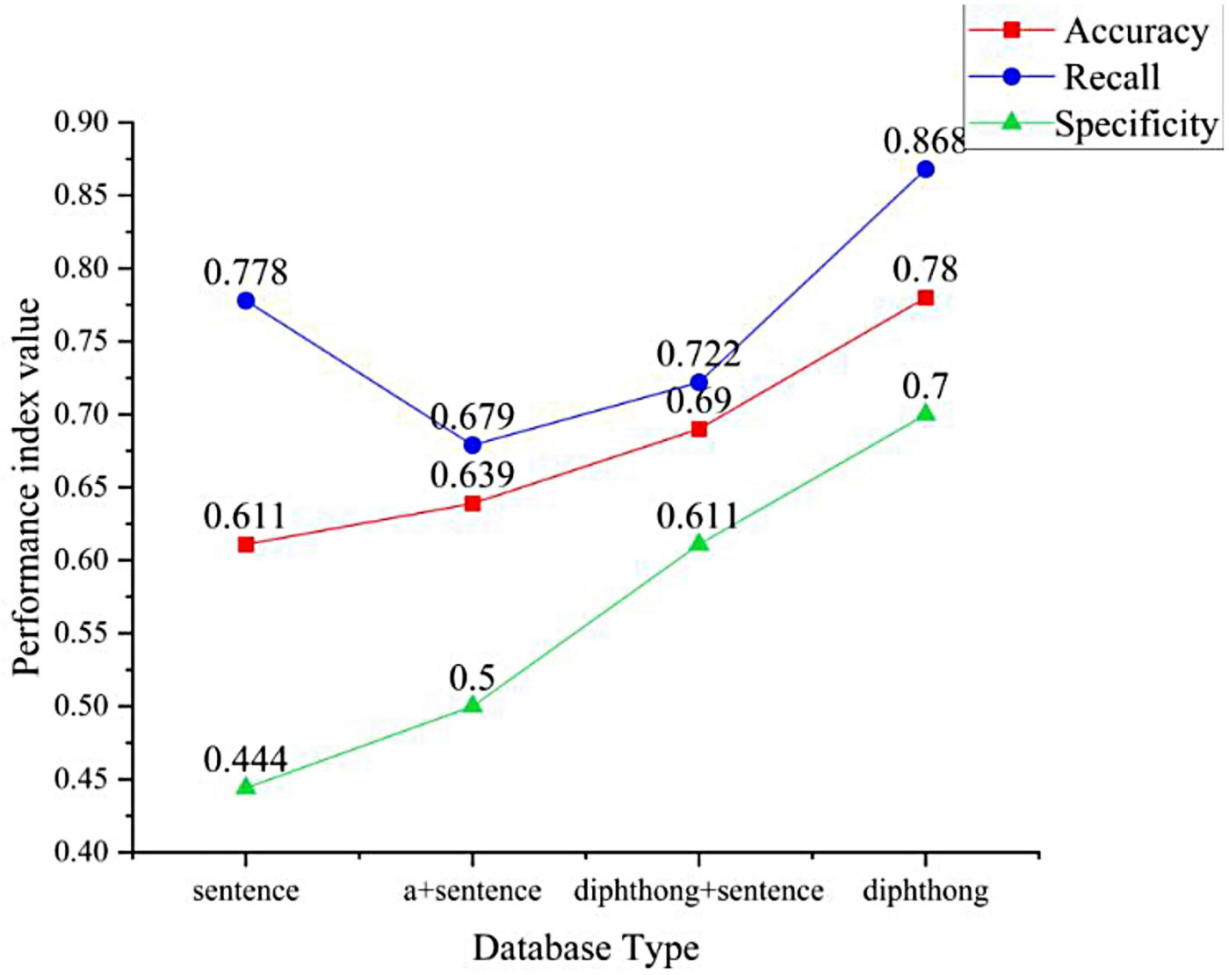
Ablation experiment results**.**

Specifically, Lasso regression, through L1 regularization, primarily retains features with strong linear correlations, effectively reducing redundancy among highly correlated acoustic parameters. In contrast, RFE employs a Random Forest classifier as the base estimator and adopts an iterative backward elimination strategy, in which features are recursively removed according to their contribution to classification performance. During this process, feature importance is evaluated based on ensemble decision trees, enabling the identification of both dominant and interaction-sensitive features.

In this study, RFE ultimately selected 6 features from the initial 14-dimensional feature space under cross-validation conditions, forming a compact yet highly discriminative subset. These features include mean fundamental frequency (meanF0), fundamental frequency variability (stdevF0), mean intensity (meanInten), intensity variability (stdevInten), local dB shimmer (localdbShimmer), and ppq5-based jitter (ppq5Jitter). From a functional perspective, meanF0 and stdevF0 characterize the baseline pitch level and its dynamic variability, reflecting neuromuscular control of vocal fold vibration. MeanInten and stdevInten capture the average vocal energy and its temporal stability, which are closely related to respiratory support and phonation control. Meanwhile, localdbShimmer and ppq5Jitter quantify cycle-to-cycle amplitude and frequency perturbations, respectively, representing micro-instabilities in vocal fold oscillation. These perturbation-related features are particularly sensitive to subtle physiological changes, such as metabolic dysfunction and neuromuscular impairment, which are commonly associated with diabetes and its complications.

Notably, the fact that RFE retained both global prosodic features (F0 and intensity statistics) and local perturbation measures indicates that the model benefits from a multi-scale representation of speech signals, capturing both macroscopic stability and microscopic irregularities. Compared with Lasso, which tends to emphasize linearly dominant features, RFE is more effective in preserving features that contribute through nonlinear interactions, thereby improving the robustness of the selected feature subset.

By merging the feature subsets obtained from Lasso and RFE, a joint feature set containing 13 key acoustic features was finally obtained (see Table 6). This hybrid strategy leverages Lasso's advantage in redundancy reduction and RFE's ability to preserve stable, discriminative, and potentially nonlinear interaction features. Furthermore, the explicit reporting of the RFE configuration (Random Forest estimator and cross-validated feature selection process) and the final selected feature subset enhances methodological transparency and ensures reproducibility. The resulting feature set provides a solid theoretical and empirical foundation for developing multidimensional speech-based diabetes detection models with improved generalization performance.

**Table 6:** Feature selection.

Feature selection methods	Selected features
Lasso	“stdevF0”, “meanInten”, “stdevInten”, “HNR”, “localShimmer”, “localdbShimmer”, “apq3Shimmer”, “apq5Shimmer”, “apq11Shimmer”, “localJitter”, “localabsJitter”, “ppq5Jitter”.
RFE	“meanF0”, “stdevF0”, “meanInten”, “stdevInten”, “localdbShimmer”, “ppq5Jitter”.
Proposed method	“meanF0”, “stdevF0”, “meanInten”, “stdevInten”, “HNR”, “localShimmer” “localdbShimmer”, “apq3Shimmer”, “apq5Shimmer”, “apq11Shimmer”, “localJitter”, “localabsJitter”, “ppq5Jitter”.

### 3.5 Identification experiment

In order to verify the superiority of the algorithm proposed in this paper, four common machine learning classification models are used to detect and identify diabe-tes in terms of performance.

The experimental results presented in Table 7 demonstrate that the proposed algorithm achieves superior and more balanced performance compared with all baseline models across multiple evaluation metrics. Specifically, the proposed Vowel-based Diabetes Type 2 (VDT) model attains the highest overall accuracy of 78.0%, outperforming K-Nearest Neighbors (KNN, 67.9%), Support Vector Machine (SVM, 62.4%), XGBoost (71.6%), and Logistic Regression (LR, 68.8%), indicating a clear advantage in overall classification capability.In terms of recall, the proposed method achieves 86.8%, which is the highest among all models and matches the performance of LR (86.8%), while significantly exceeding KNN (66.2%), SVM (61.8%), and XGBoost (75.0%). This demonstrates that the proposed model is highly effective in identifying diabetic patients and minimizing missed diagnoses, which is critical in medical screening tasks.

**Table 7:** Recognition experiment results.

Model	Accuracy (%)	Recall (%)	Specificity (%)	AUC
KNN	0.679	0.662	0.707	0.74
SVM	0.624	0.618	0.634	0.69
XGBoost	0.716	0.750	0.659	0.80
LR	0.688	0.868	0.390	0.68
VDT (This algorithm)	0.780	0.868	0.700	0.79

However, a more detailed comparison reveals that LR, despite its high recall, suffers from extremely low specificity (39.0%), indicating a high false-positive rate and limited practical applicability. In contrast, the proposed method achieves a specificity of 70.0%, substantially higher than LR (39.0%) and also outperforming XGBoost (65.9%), KNN (70.7% comparable), and SVM (63.4%). This indicates that the proposed model not only maintains high sensitivity but also effectively reduces false positives, achieving a more favorable balance between sensitivity and specificity.

Regarding the AUC metric, the proposed model achieves 0.79, which is comparable to XGBoost (0.80) and significantly higher than KNN (0.74), SVM (0.69), and LR (0.68). Although XGBoost slightly outperforms the proposed model in AUC, it does not achieve the same level of balance between recall and specificity, as its recall (75.0%) is notably lower.

Overall, the proposed algorithm demonstrates a clear advantage in achieving a well-balanced trade-off among accuracy, recall, and specificity. This balanced performance is particularly important in clinical screening scenarios, where both high sensitivity (to avoid missed diagnoses) and adequate specificity (to reduce unnecessary follow-up examinations) are essential. These results highlight the robustness, reliability, and practical applicability of the proposed method for speech-based diabetes detection.

### 3.6 Comparative experiment

To further verify the role of vowels in diabetes detection, we designed a cor-pus-based ablation experiment and compared four speech input forms:

1. Sentence (29): Standardized reading of “Mom has a green jade pot’;
2. Monophthongs /a/ + sentence (30): Standardized reading of /a/ + “Mom has a green jade pot”;
3. Polyphonic sounds /a/, /o/, /e/, /i/, /u/, /ü/ + sentence: Standardized reading of /a/, /o/, /e/, /i/, /u/, /ü/ + “Mom has a green jade pot”;
4. Polyphonic sounds /a/, /o/, /e/, /i/, /u/, /ü/: Standardized reading of /a/, /o/, /e/, /i/, /u/, /ü/;

The experimental results in Table 8 show that replacing a single vowel with multiple vowels improved the model's accuracy from 63.9% to 69.0% and specificity from 50.0% to 61.1%. This indicates that multiple vowels provide richer acoustic information, enabling the model to capture more comprehensive speech features related to diabetes. However, the AUC value decreased slightly, from 0.64 to 0.63, which may be due to the generalization challenges posed by the complex combination of vowels and sentence structure. More notably, when semantic information was completely removed and only multiple vowels were used as input, the model performance significantly improved: accuracy increased to 78.0%, recall (sensitivity) reached 86.8%, specificity increased to 70.0%, and the AUC value reached 0.79, significantly outperforming the previous two methods. The visualization results are shown in Figure 10. These findings demonstrate that vowels alone, without considering contextual semantics, contain key acoustic differences capable of distinguishing between diabetic patients and healthy individuals, thus enabling a high-performance automatic recognition model. Therefore, vowels can be considered to play a central role in speech recognition in diabetes.

**Table 8:** Ablation experiment results.

Database type	Accuracy (%)	Recall (%)	Specificity (%)	AUC
Sentence	0.611	0.778	0.444	0.359
/a/+ Sentence	0.639	0.679	0.500	0.64
/a/, /o/, /e/, /i/, /u/, /ü/+ Sentence	0.690	0.722	0.611	0.63
/a/, /o/, /e/, /i/, /u/, /ü/	0.780	0.868	0.700	0.79

## 4 Discussion

Although vowel-based diabetes detection technology has made initial progress in studies using Chinese corpora, several challenges remain. First, due to current limitations in expertise and technical resources, this study was unable to conduct an in-depth analysis of the specific roles played by the six types of Chinese vowels in diabetes detection, which represents a core focus for future research.Second, the limited sample size of the available Chinese speech database and the relative scarcity of high-quality annotated speech data significantly constrain the model's learning capacity. In particular, small sample sizes may lead to overfitting, where the model captures dataset-specific patterns rather than generalizable acoustic biomarkers, thereby reducing its robustness when applied to unseen populations or external datasets. As a result, the generalization performance of the proposed model is inevitably affected under small-sample conditions, highlighting the need for larger and more diverse datasets in future work.Finally, the subtle and concealed nature of early diabetes symptoms and its complications often produces weak and difficult-to-detect acoustic signal alterations, making significant improvements in sensitivity and detection accuracy another key technical challenge.

## Data Availability

The raw data supporting the conclusions of this article will be made available by the authors, without undue reservation.
